# New approach to type B aortic dissection – zone 2.5 thoracic endovascular aortic repair: a case report

**DOI:** 10.1186/s13019-025-03828-6

**Published:** 2026-01-03

**Authors:** Hitoki Hashiguchi, Naomi Yasuda, Akihito Ohkawa

**Affiliations:** 1 Department of Cardiovascular Surgery, Hokkaido Prefectural Kitami Hospital, Kitami Hokkaido, 090-0027 Japan; 2 Department of Cardiovascular Surgery, Sapporo Medical University School of Medicine, Sapporo Hokkaido, 060-8556 Japan

**Keywords:** Type B aortic dissection, Remodeling, TEVAR

## Abstract

We report a novel thoracic endovascular aortic repair (TEVAR) technique for complicated type B aortic dissection requiring zone 2 landing. A 42-year-old man presented with pulseless left femoral artery. Emergency TEVAR was performed using the left subclavian artery (LSA) wall as a sealing zone to reduce the risk of endoleak. Postoperative recovery was uneventful, and the patient was discharged on day 8. CT demonstrated complete coverage of the entry tear, false lumen thrombosis, and preserved left subclavian perfusion. Follow-up CT showed favorable remodeling. This technique may be effective in patients with limited proximal sealing zones.

**Supplementary Information:**

The online version contains supplementary material available at 10.1186/s13019-025-03828-6.

## Introduction

Thoracic endovascular aortic repair (TEVAR) is the optimal therapy of care for complicated type B aortic dissection (TBAD) [1–5]. Approximately 20% of TEVAR for TBAD requires covering the left subclavian artery (LSA) for the proximal sealing zone [3, 6]. Some cases of TEVAR require a zone 2 landing to cover the primary entry of the dissection. These cases require protection of the LSA’s circulation. Many strategies have been used for LSA protection, including branched TEVAR, chimney technique, and bypass for LSA [2, 7–9]. Although short-term morbidity and mortality are low, TEVAR has subsequent complications such as the risk of endoleaks and device migration, especially in those with revascularized LSA [6, 8, 9].

We report the successful treatment of TBAD with a new approach in TEVAR. This technique, termed Zone 2.5 TEVAR, was designed to extend the proximal landing zone distally toward the origin of the LSA without performing LSA revascularization. Unlike the conventional “half-coverage” technique—which aims to extend the sealing zone proximally into the aortic arch—Zone 2.5 TEVAR defines an intermediate landing area between classical Zones 2 and 3. By partially using the LSA wall as a sealing zone, this approach achieves a sufficient proximal seal while avoiding excessive manipulation within the arch and reducing the risk of retrograde type A dissection.

## Case report

**Fig. 1:**
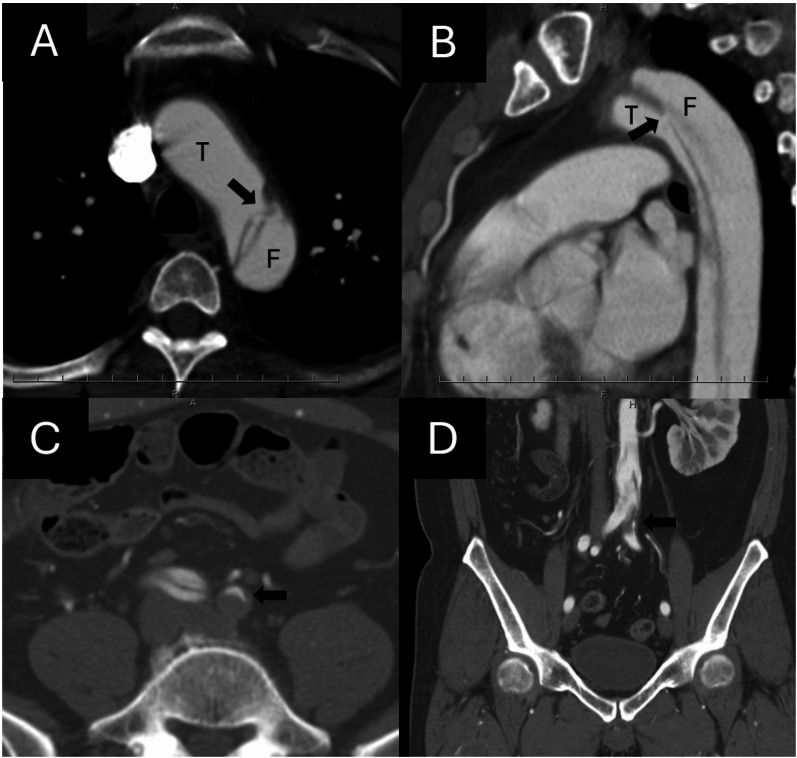
Preoperative contrast-enhanced computed tomography (CT) images. (**A**,** B**) Axial and sagittal views at the aortic arch level show the primary entry tear (black arrows) located just distal to the origin of the left subclavian artery (LSA). (**C**,** D**) Axial and coronal views at the level of the iliac bifurcation demonstrate collapse of the left common iliac artery (arrows) due to compression of the true lumen by the false lumen

A 42-year-old man with a history of acute myocardial infarction several years ago was being followed at a nearby hospital. The patient was a current smoker, and his medical history included hypertension, dyslipidemia, and hyperuricemia. He presented with acute chest pain at a nearby hospital. Initially, he was diagnosed with cardiac angina, and nitroglycerin medications were administered. Computed tomography (CT) revealed TBAD. He was transferred to our hospital at 3 AM the next day owing to continuous chest pain. On arrival at our hospital, the chest pain had disappeared; however, the left limb pain worsened and the left limb became pale. Computed tomography (CT) revealed type B aortic dissection (TBAD) with the primary entry tear located just distal to the origin of the left subclavian artery (LSA) (Fig. 1A, B). At the level of the iliac bifurcation, the left common iliac artery was completely compressed by the false lumen, resulting in left lower limb ischemia (Fig. 1C, D). The distance from the LSA to the primary entry tear was 8 mm, and emergency TEVAR was performed.

General anesthesia was induced. The left brachial artery was accessed with a 5Fr Glidesheath Slender (Terumo Corporation) for angiography. The left femoral artery was cannulated under ultrasound guidance with a 9Fr sheath. IVUS confirmed true lumen wire placement. Two Perclose ProGlide sutures (Abbott Vascular) were placed at 10:00 and 2:00 o’clock, and a 9Fr sheath was inserted. A Radifocus Guidewire (Terumo Corporation) was exchanged for a Lunderquist extra stiff wire (Cook Medical Inc.).

**Fig. 2:**
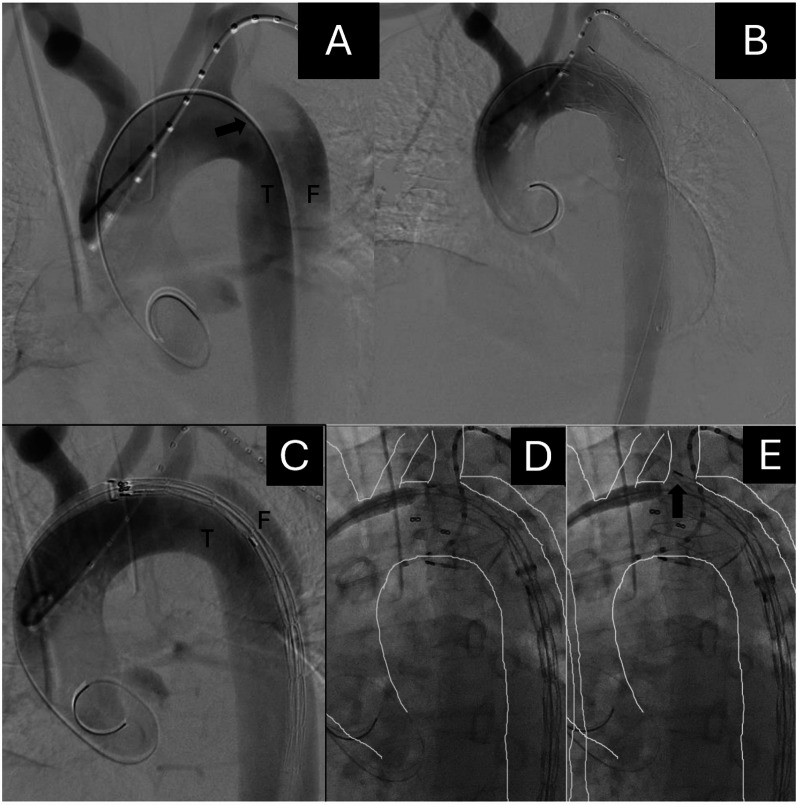
Intraoperative angiography during thoracic endovascular aortic repair (TEVAR). (**A**) Initial aortogram shows the primary entry tear (black arrow) located just distal to the origin of the LSA. True (T) and false (F) lumens are indicated. (**B**) Completion angiography after stent-graft deployment demonstrates that the proximal edge of the stent graft is positioned slightly within the LSA lumen, achieving a stable proximal seal (Zone 2.5 landing) with preserved LSA perfusion. (**C–E**) Sequential angiographic views during deployment. (**C**) Aortogram obtained immediately before stent-graft deployment. (**D**) The greater-curvature portion of the proximal stent approaches the LSA orifice, and (**E**) the greater-curvature edge of the stent graft enters the LSA lumen (arrow), serving as a practical landmark for achieving the intended Zone 2.5 landing. The white tracing line outlines the aortic contour to clarify the spatial relationship between the aorta and the stent graft

A pigtail catheter was advanced into the ascending aorta through the left radial artery for angiography, confirming the origins of the BCA, left carotid, and LSA (Figure 2A). A VALIANT Thoracic Stent Graft with the Captivia Delivery System (30 × 26 mm, 150 mm, Closed Web; Medtronic) was used. Blood pressure was maintained below 90 mmHg. Sequential intraoperative angiography demonstrated the deployment process, showing the greater-curvature edge of the proximal stent entering the LSA lumen to achieve the intended Zone 2.5 landing (Fig. 2C–E). Completion angiography confirmed exclusion of the false lumen and preserved LSA perfusion (Fig. 2B). The first stent was fixed at this position, and subsequent deployment achieved the planned Zone 2.5 landing.

**Fig. 3:**
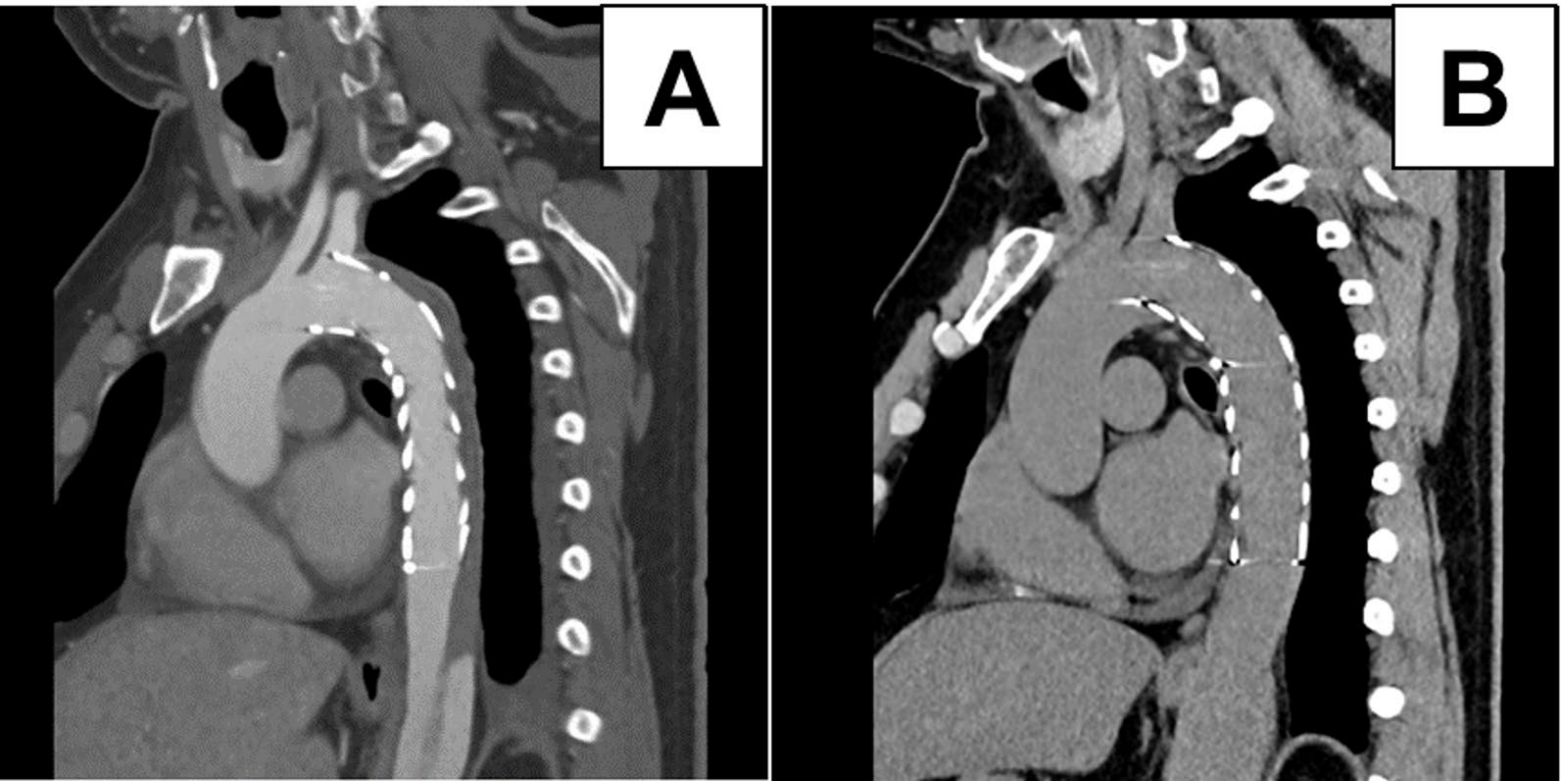
Postoperative contrast-enhanced computed tomography (CT) images after TEVAR. (**A**) CT obtained 4 days postoperatively shows complete exclusion of the primary entry tear and thrombosis of the false lumen in the thoracic aorta. (**B**) CT at 1 year follow-up demonstrates sustained false lumen thrombosis and favorable aortic remodeling with preserved LSA perfusion

Angiography showed complete exclusion of the false lumen and improved left limb perfusion. The operation time was 50 min. The patient’s postoperative course was uneventful. Postoperative CT confirmed complete coverage of the primary entry and thrombosis of the false lumen (Fig. 3A). At 1-year follow-up, CT showed sustained false lumen thrombosis and favorable aortic remodeling with preserved LSA perfusion (Fig. 3B). An intraoperative video showing the deployment process is provided as additional material (Supplementary Video 1).

## Discussion

We report a patient with acute TBAD successfully treated with a new approach. TEVAR is recognized as the standard therapy for complicated TBAD and has been shown to decrease mortality compared to open surgery by promoting true lumen expansion and aortic remodeling [4, 5]. However, false lumen patency remains a challenge requiring secondary interventions [6]. Achieving a dissection-free proximal sealing zone often necessitates LSA coverage. Conventional strategies such as debranching, fenestrated grafts, or physician-modified endografts permit more proximal coverage but can delay definitive repair or carry procedural risk [7, 8]. The chimney technique allows revascularization but is associated with higher rates of type I endoleak from gutters or proximal stent compression [8, 9].

This new procedure is advantageous because additional steps such as debranching or fenestration are unnecessary, operation time is shorter, and no extra prosthesis is required. Using the LSA as part of the sealing zone effectively lengthens the seal and may reduce the risk of type I endoleak and incomplete entry coverage. Anchoring the proximal portion of the stent graft against the LSA wall also stabilizes the device and prevents distal migration.

As shown in Fig. 2, the greater-curvature edge of the stent graft was intentionally advanced slightly into the LSA lumen, serving as a reproducible landmark for the Zone 2.5 landing. Conceptually, the Zone 2.5 TEVAR technique differs from previously described partial-coverage or “half-coverage” methods. Its purpose is to extend the proximal landing zone distally toward the LSA origin, thereby securing an adequate sealing length without advancing the stent graft further into the arch. The greater-curvature edge of the stent graft is intentionally positioned slightly within the lumen of the LSA, serving as a practical landmark for optimal proximal positioning. This controlled placement provides stable fixation and preserves LSA perfusion without the need for revascularization. Since this first case, the Zone 2.5 concept has been applied in more than 30 dissection cases at our institution and has been adopted by several other high-volume centers in Japan, underscoring its reproducibility and clinical relevance.

## Conclusion

We describe a case of acute TBAD successfully treated using the Zone 2.5 TEVAR approach. This strategy extends the proximal landing zone distally toward the LSA, providing an effective sealing length without the need for revascularization. It may represent a reproducible and minimally invasive option for patients with short proximal landing zones.

## Supplementary Information

Supplementary Material 1

## Data Availability

No datasets were generated or analysed during the current study.

## Abbreviations

TEVAR: Thoracic endovascular aortic repair
TBAD: Type B aortic dissection
LSA: Left subclavian artery
CT: Computed tomography
